# Apparent diffusion coefficient and tissue stiffness are associated with different tumor microenvironment features of hepatocellular carcinoma

**DOI:** 10.1007/s00330-024-10743-2

**Published:** 2024-05-20

**Authors:** Jie Chen, Zhenru Wu, Zhen Zhang, Yidi Chen, Meng Yin, Richard L. Ehman, Yuan Yuan, Bin Song

**Affiliations:** 1 Department of Radiology, West China Hospital, Sichuan University, No. 37 Guoxue Alley, Chengdu, 610041 China; 2 Department of Radiology, Functional and Molecular Imaging Key Laboratory of Sichuan Province, West China Hospital, Sichuan University, Chengdu, 610041 China; 3 Laboratory of Pathology, West China Hospital, Sichuan University, No. 88 South Keyuan Road, Chengdu, 610041 China; 4 Department of Radiology, Mayo Clinic, Rochester, MN 55905 USA

**Keywords:** Carcinoma, hepatocellular, Diffusion magnetic resonance imaging, Magnetic resonance elastography, Cell density, Tumor microenvironment

## Abstract

**Objectives:**

To investigate associations between tissue diffusion, stiffness, and different tumor microenvironment features in resected hepatocellular carcinoma (HCC).

**Methods:**

Seventy-two patients were prospectively included for preoperative magnetic resonance (MR) diffusion-weighted imaging and MR elastography examination. The mean apparent diffusion coefficient (ADC) and stiffness value were measured on the central three slices of the tumor and peri-tumor area. Cell density, tumor-stroma ratio (TSR), lymphocyte-rich HCC (LR-HCC), and CD8 + T cell infiltration were estimated in resected tumors. The interobserver agreement of MRI measurements and subjective pathological evaluation was assessed. Variables influencing ADC and stiffness were screened with univariate analyses, and then identified with multivariable linear regression. The potential relationship between explored imaging biomarkers and histopathological features was assessed with linear regression after adjustment for other influencing factors.

**Results:**

Seventy-two patients (male/female: 59/13, mean age: 56 ± 10.2 years) were included for analysis. Inter-reader agreement was good or excellent regarding MRI measurements and histopathological evaluation. No correlation between tumor ADC and tumor stiffness was found. Multivariable linear regression confirmed that cell density was the only factor associated with tumor ADC (Estimate = −0.03, *p* = 0.006), and tumor-stroma ratio was the only factor associated with tumor stiffness (Estimate = −0.18, *p* = 0.03). After adjustment for fibrosis stage (Estimate = 0.43, *p* < 0.001) and age (Estimate = 0.04, *p* < 0.001) in the multivariate linear regression, intra-tumoral CD8 + T cell infiltration remained a significant factor associated with peri-tumor stiffness (Estimate = 0.63, *p* = 0.02).

**Conclusions:**

Tumor ADC surpasses tumor stiffness as a biomarker of cellularity. Tumor stiffness is associated with tumor-stroma ratio and peri-tumor stiffness might be an imaging biomarker of intra-tumoral immune microenvironment.

**Clinical relevance statement:**

Tissue stiffness could potentially serve as an imaging biomarker of the intra-tumoral immune microenvironment of hepatocellular carcinoma and aid in patient selection for immunotherapy.

**Key Points:**

- *Apparent diffusion coefficient reflects cellularity of hepatocellular carcinoma*.
- *Tumor stiffness reflects tumor-stroma ratio of hepatocellular carcinoma and is associated with tumor-infiltrating lymphocytes*.
- *Tumor and peri-tumor stiffness might serve as imaging biomarkers of intra-tumoral immune microenvironment*.

## Introduction

Hepatocellular carcinoma (HCC) is the sixth most prevalent cancer and the second leading cause of cancer-related deaths worldwide [1]. Magnetic resonance imaging (MRI) plays an essential role in the diagnosis and local staging of HCC. However, beyond its great diagnostic capacity, MRI also provides valuable insights into the histopathology of HCC, which holds significant clinical implications.

One of the key metrics derived from MRI is the apparent diffusion coefficient (ADC), which is quantified from diffusion-weighted imaging (DWI), a commonly used non-contrast quantitative MRI technique. Accumulating evidence suggests a correlation between ADC and several critical histopathological features of HCC, including tumor differentiation, proliferation, and microvascular invasion [2–5]. A recent study further highlighted a weak association between ADC and tumor-stroma ratio (STR) in HCC [6], which is considered a crucial microenvironment feature of HCC and might be associated with treatment response and patient prognosis [7, 8]. Additionally, ADC values have also been linked to the density of tumor-infiltrating lymphocytes in several malignancies such as head and neck squamous cell cancer, breast cancer, and intrahepatic cholangiocarcinoma [9–11]. However, the exploration of such a relationship in the context of HCC remains unclear.

Another MRI technique gaining prominence in HCC assessment is magnetic resonance elastography (MRE). MRE provides a non-invasive non-contrast method for quantifying tissue stiffness, a biomechanical property associated with extracellular matrix deposition and a key parameter in the evaluation of liver fibrosis [12]. Prior studies have shown that tumor stiffness was associated with the presence of microvascular invasion (MVI), response to transarterial chemoembolization treatment and immunotherapy, and tumor recurrence [13–15]. Understanding the relationship between tumor stiffness and tumor microenvironments, such as cellularity, TSR, and immune microenvironment, could potentially enhance patient stratification and guide personalized management strategies using MRI-derived information. However, little has been known about the relationship between tumor stiffness and histopathology in HCC.

Therefore, the purpose of this study was to investigate possible associations between ADC, MRE-derived stiffness, and different histopathological features including cell density, TSR, tumor-infiltrating lymphocytes, and CD8 + T cell infiltration in resected HCC.

## Materials and methods

### Study population

This prospective study was approved by our institutional review board and written informed consent was obtained from each participant. From January 2021 to March 2023, patients suspected with HCC were recruited for preoperative MRI/MRE examination. The inclusion criteria for initial patient recruitment included patients with liver lesions suspected of HCC detected by other imaging modalities (ultrasonography or computed tomography) and/or elevated serum tumor markers (Alpha-fetoprotein and/or Protein Induced by Vitamin K Absence or Antagonist-II). The final exclusion criteria were: (a) patients with pathology-proven non-HCC lesions, (b) pathology from surgically resected tumor samples within one month after the MRI examination was not available, (c) patients received local regional or systemic antitumoral therapy before MRI examination, (d) tumor diameter was less than 1 centimeter, (e) tumor located at the hepatic dome, (f) mechanical coupling failure due to a loose driver, and (g) technical failure due to severe respiratory motion artifacts. Demographic information and laboratory results were collected from the electronic medical system.

### MRI examination

All MRI examinations were performed prospectively on a 3.0 T MR scanner (Discovery MR750, GE Healthcare) using a 16-channel phased-array body coil. Participants were fasted at least 4 h before the MRI scan. Routine axial DWI was scanned under free breath using the following parameters: TR = 6315 msec, TE = 70.7 msec, FOV = 400 × 320 mm, matrix = 80 × 128, slice thickness = 6.0 mm, b values = 0, 50, and 1000 sec/mm^2^, and acceleration factor = 2. Apparent diffusion coefficient (ADC) was calculated from b values of 0 and 1000 sec/mm^2^ by using the mono-exponential model.

MRE examination was conducted after a routine pre-scan and before the application of the contrast agent. For liver MRE, a customized flexible acoustic passive driver (30 × 10 cm, Mayo Clinic) was placed over the right upper abdomen and was secured using an elastic strap. 3D-MRE data was acquired during breath hold at the end of expiration by a single-shot echo-planar imaging sequence using the following parameters: TR = 1600 msec, TE = 57.8 msec, FOV = 440 × 440 mm, matrix = 96 × 96, slice thickness = 3.6 mm, mechanical vibration frequency = 60 Hz, and acceleration factor = 2. Post-processing of 3D-MRE was conducted by using a 3D multimode direct inversion algorithm to generate corresponding wave images, phase images, magnitude images, and shear stiffness maps.

### Image analysis

Image analysis was conducted using the ITK-SNAP software (Version 3.6.0) by two independent investigators (C.J. and Z.Z., with 10- and 6-years’ experience in liver MRI, respectively) who were blinded to histopathological findings. By using T2-weighted and contrast-enhanced images as references, the freehand region of interest was manually drawn on the central three slices of the tumor on the stiffness and ADC map to encircle the tumor area. Tumor edges and areas with necrosis, intra-tumoral hemorrhage, artifacts, and poor wave penetration, as determined by the investigator, were excluded from the analysis. Peri-tumor stiffness and peri-tumor ADC were quantified by placing freehand regions of interest on the same three slices on peri-tumor liver parenchyma (within 1 cm from the tumor edge), while excluding artifacts and major vasculatures. When multiple lesions were detected, the lesion with the largest diameter was selected for imaging and pathological analysis. The volumetric mean value of each parameter was extracted, and the final measurement was calculated as the average value of two measurements.

### Histopathological analysis

Tumor specimen was obtained from the tumor center section following surgical resection, as per the prior recommendation [16]. For each specimen, a standard hematoxylin and eosin (HE) staining and a multiplex immunohistochemical (mIHC) staining were performed. Histopathological findings were evaluated in the scanned whole slide image using the open-source software of QuPath (Version 0.4.3). For quantitative evaluation of cell density and TSR, a pathologist (W.Z., 10 years’ experience in liver pathology) first reviewed the 5-channal mIHC slide and a free hands annotation was defined to include all available tumor area for quantitative analysis. The watershed cell detection method was then applied to detect and segment all cells based on the nucleus stain. Following a similar approach to previous studies, TSR was quantified on a cellular level using machine learning-based methods in Qupath [17–19] (detailed information is provided in supplementary methods). Cell density was calculated as the total number of cells divided by the annotation area at a low power field of 4X on mIHC slides, and TSR was calculated as the total number of tumor cells divided by the total number of stroma cells based on cell detection and classification on mIHC slides, as described in supplementary methods.

Additionally, a qualitative assessment of tumor-infiltrating lymphocytes and CD8 + T cells was conducted by two independent investigators (W.Z. and C.J.), and a consensus-reached categorization was used for analysis. Lymphocytes-rich HCC (LR-HCC) was defined as HCC with a diffuse dense intra-tumoral lymphocytes infiltration and involving more than 50% of the tumor on HE slides [20]. CD8 + T cell infiltration was categorized as negative (none or mild) and positive (moderate or dense) based on mIHC slides. Other histopathological features such as the Edmondson-Steiner grade, MVI, the fibrosis stage, and inflammation grade of peri-tumor liver parenchyma were also assessed by the pathologist.

### Statistical analysis

The interobserver agreement was assessed using the intraclass correlation coefficient (ICC) for quantitative MRI measurements and Cohen’s Kappa for subjective pathological evaluation. The correlation between imaging biomarkers and histopathological features was calculated by Spearman’s rank correlation coefficient. Variables influencing ADC and stiffness were screened with univariable analyses, and then identified with multivariable linear regression. The potential relationship between explored imaging biomarkers and histopathological features was assessed with linear regression after adjustment for other influencing factors. Differences in imaging and histopathological biomarkers were compared between HCCs with and without MVI using the Mann-Whitney test or independent-sample *T*-test when appropriate. Statistical analysis was performed using the SPSS software (IBM SPSS Statistics for Windows, version 28: IBM corporation, 2021) and R software (R version 4.3.1). A significance level of *p* < 0.05 was used to determine statistical significance.

## Results

**Fig. 1:**
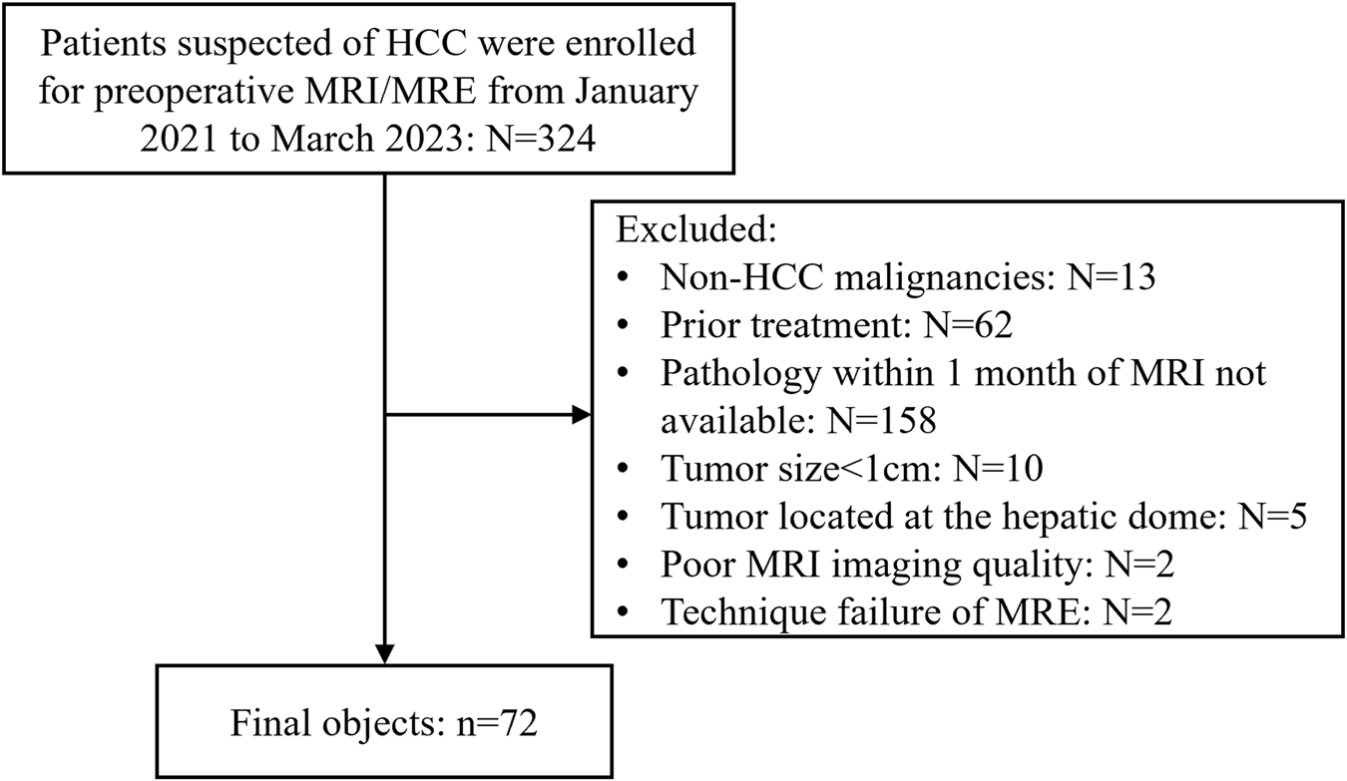
Flow chart of patient selection

The final study cohort included 72 patients (male/female: 59/13, mean age: 56 ± 10.2 years), sixty-three of them had hepatitis B virus infection. Among them, 57 patients had solitary lesion, 9 patients had 2 lesions, and 6 patients had 3 lesions. The distribution of the Barcelona Clinic Liver Cancer (BCLC) stage was 54 in stage A, 12 in stage B, and 6 in stage C. All patients received liver resection in accordance with the guideline recommendation for BCLC stage A or the expanded criteria for selected patients with BCLC stage B or C (preserved liver function, no more than 3 nodules, or portal invasion to the first-, second- and third-order branch). Macrovascular invasion was present in 6 patients, while 30 patients had MVI. The median tumor diameter was 3.70 cm. Figure 1 represents the flowchart of this study. Demographic, clinical, and pathological characteristics of included patients were summarized in Table 1.

**Table 1:** Demographic, clinical, radiological, and histopathological characteristics of included patients

Characteristics	Results
Male/Female (*n*)	59/13
Age (years)	56 ± 10.2
Hepatitis B virus infection	63/72
Alpha-fetoprotein (ng/mL)	12.95 (3.38, 494.00)
PIVKA_II (mAU/mL)	217.50 (53.00, 2293.00)
Carbohydrate antigen 19-9 (U/mL)	17.85 (10.60, 26.80)
Alanine aminotransferase (IU/L)	28.50 (20.00, 51,00)
Aspartate aminotransferase (IU/L)	30.00 (23.00, 44.50)
Albumin (g/L)	43.20 (41.30, 46.55)
Total bilirubin (μmol/L)	14.70 (10.55, 17.10)
Indirect bilirubin (μmol/L)	9.20 (6.80, 12.40)
Direct bilirubin (μmol/L)	4.55 (3.70, 5.70)
Platelet count (×10^9^/L)	146.00 (92.00, 183.00)
Tumor diameter (cm)	3.70 (2.25, 5.25)
BCLC stage (A/B/C)	54/12/6
Cirrhosis (Yes/No)	62/10
Macrovascular invasion (Yes/No)	6/66
Edmondson-Steiner Grade (I/II/III)	4/50/18
Microvascular invasion (Yes/No)	30/42
Tumor ADC (sec/mm^2^)	1.12 ± 1.34
Tumor stiffness (kPa)	4.52 ± 1.48
Peri-tumor ADC (mm^2^/s)	1.34 ± 0.26
Peri-tumor stiffness (kPa)	3.34 ± 1.08
Cell density (×10^3^/mm^2^)	10.09 ± 2.93
Tumor-stroma ratio	3.23 ± 2.42

Data in parenthesis are quartiles

*PIVKA-II* Protein induced by vitamin K absence or antagonist-II, *BCLC stage* the Barcelona Clinic Liver Cancer stage

### Association between ADC value and stiffness

**Fig. 2:**
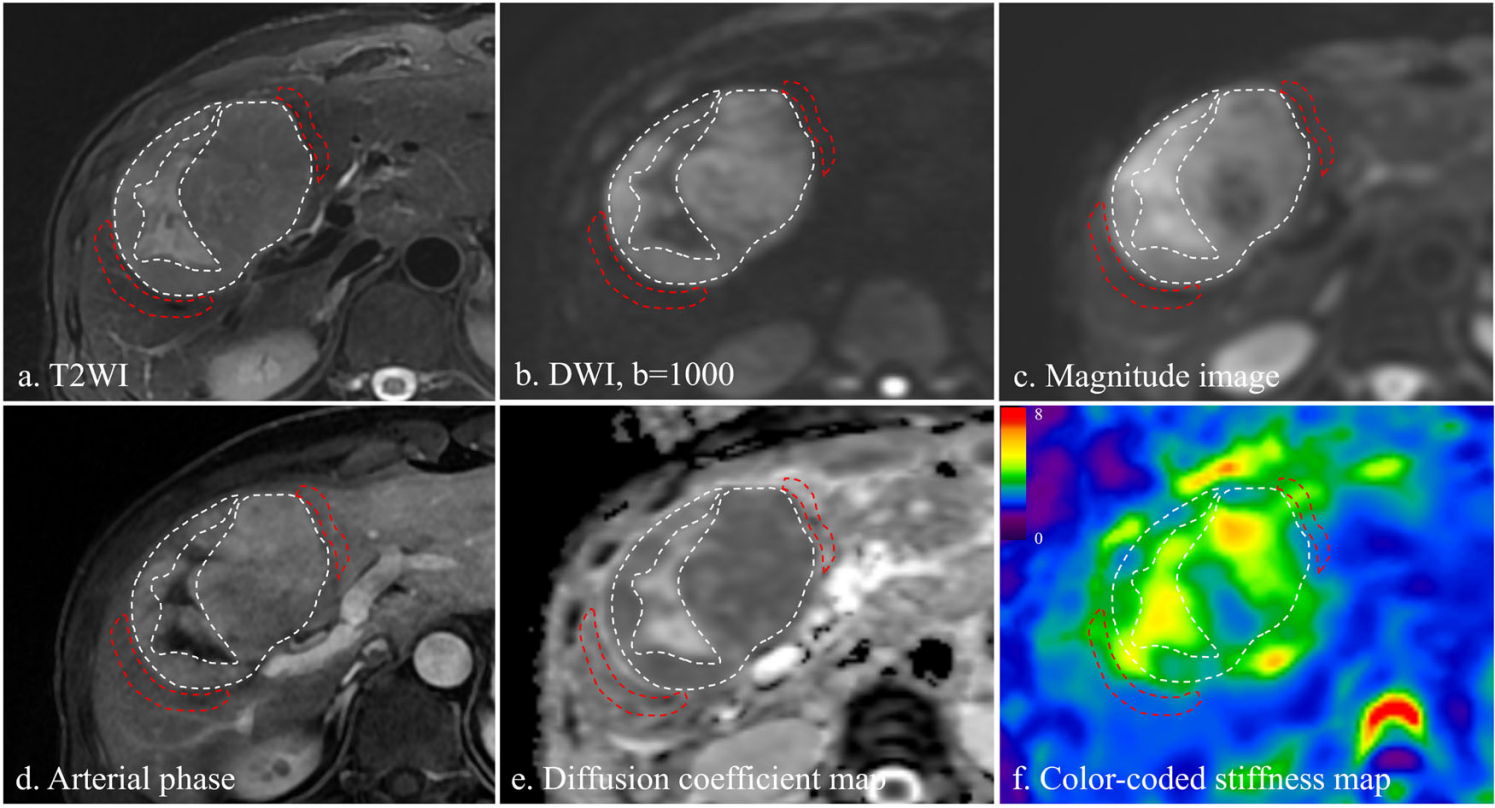
The measurement of apparent diffusion coefficient and stiffness in the tumor (white) and peri-tumor area (red). **a** T2 weighted image; **b** diffusion-weighted image; **c** the T2-weighted magnitude image of magnetic resonance elastography; **d** late arterial phase of dynamic contrast enhanced imaging; **e** the apparent diffusion coefficient map; **f** the color-coded stiffness map

Inter-reader agreement was good or excellent regarding ADC and stiffness measurement. The ICC was 0.82 (0.69, 0.90) for tumor ADC, 0.94 (0.87, 0.98) for tumor stiffness, 0.75 (0.57, 0.91) for peri-tumor ADC, and 0.95 (0.84, 0.99) for peri-tumor stiffness. The mean tumor ADC was 1.12 ± 1.34 mm^2^/s, mean tumor stiffness was 4.52 ± 1.48 kPa, mean peri-tumor ADC was 1.34 ± 0.26 mm^2^/s, and mean peri-tumor stiffness was 3.34 ± 1.08 kPa. Figure 2 demonstrates the measurement of imaging biomarkers, more cases with MRI images are provided in Supplementary Figs. 1, 2, and 3. The influence of intra-tumoral necrosis on the agreement and measurement of each variable was summarized in Supplementary Table 1 and Table 2.

**Table 2:** Associations between ADC value and stiffness in the tumor and peri-rumor parenchyma

Imaging biomarkers	Tumor stiffness	Peri-tumor ADC	Peri-tumor stiffness
Tumor ADC	0.08 (0.54)	**0.49 (< 0.001)**	−0.06 (0.65)
Tumor stiffness		−0.11 (0.38)	**0.50 (< 0.001)**
Peri-tumor ADC			**−0.35 (0.003)**

Data in parenthesis are *p* values, and significant correlations are given in boldface

*ADC* apparent diffusion coefficient

**Fig. 3:**
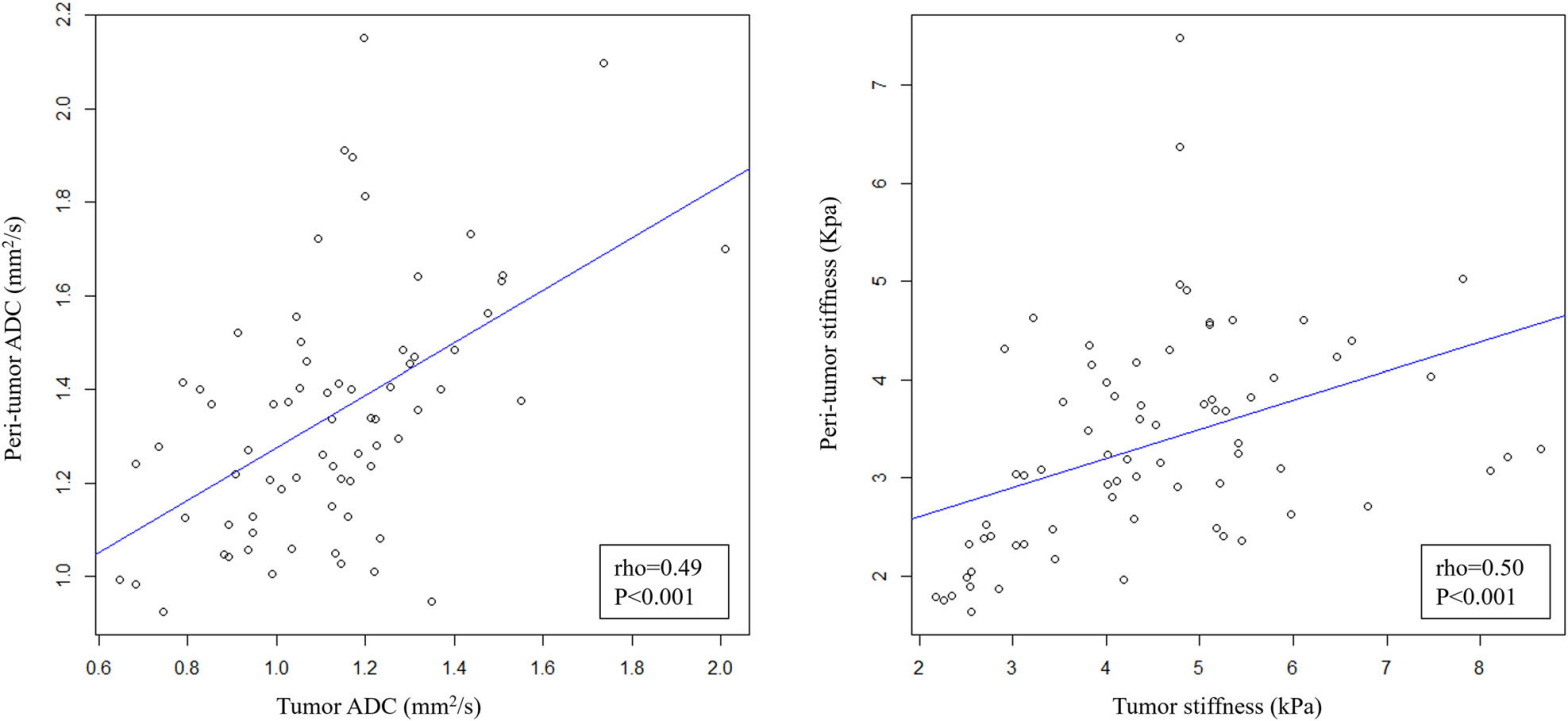
The associations between tumor and peri-tumor MRI measurements in hepatocellular carcinoma

There were significant correlations between tumor ADC and peri-tumor ADC (rho = 0.49, *p* < 0.001), between tumor stiffness and peri-tumor stiffness (rho = 0.50, *p* < 0.001), and between peri-tumor ADC and peri-tumor stiffness (rho = −0.35, *p* = 0.003). However, no significant correlation was found between tumor ADC and tumor stiffness (rho = 0.08, *p* = 0.54) (Table 2). Figure 3 demonstrates the association between tumor and peri-tumor measurements.

### Association between different histopathological features

**Fig. 4:**
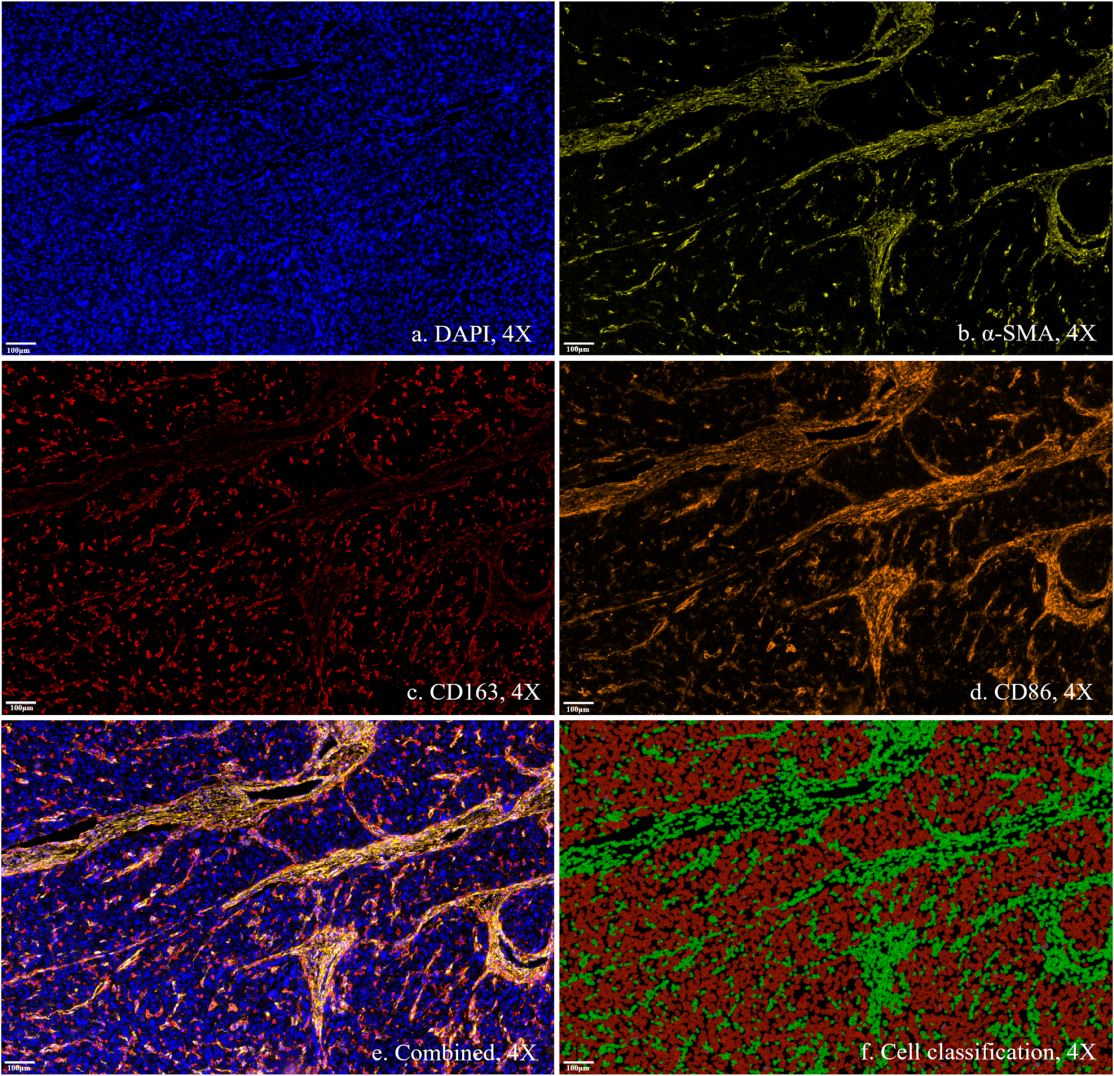
Demonstration of each biomarker and their combined expression on the multiplex immunohistochemical stain, along with the results of cell classification. **a** The nucleus stain by 4',6-diamidino-2-phenylindole (DAPI). **b** The expression of α-smooth muscle actin (α-SMA). **c** The expression of CD163. **d** The expression of CD86. **e** Combined expression of multiple biomarkers. **f** Results of cell classification. Red indicates tumor cells and green indicates stroma cells

**Fig. 5:**
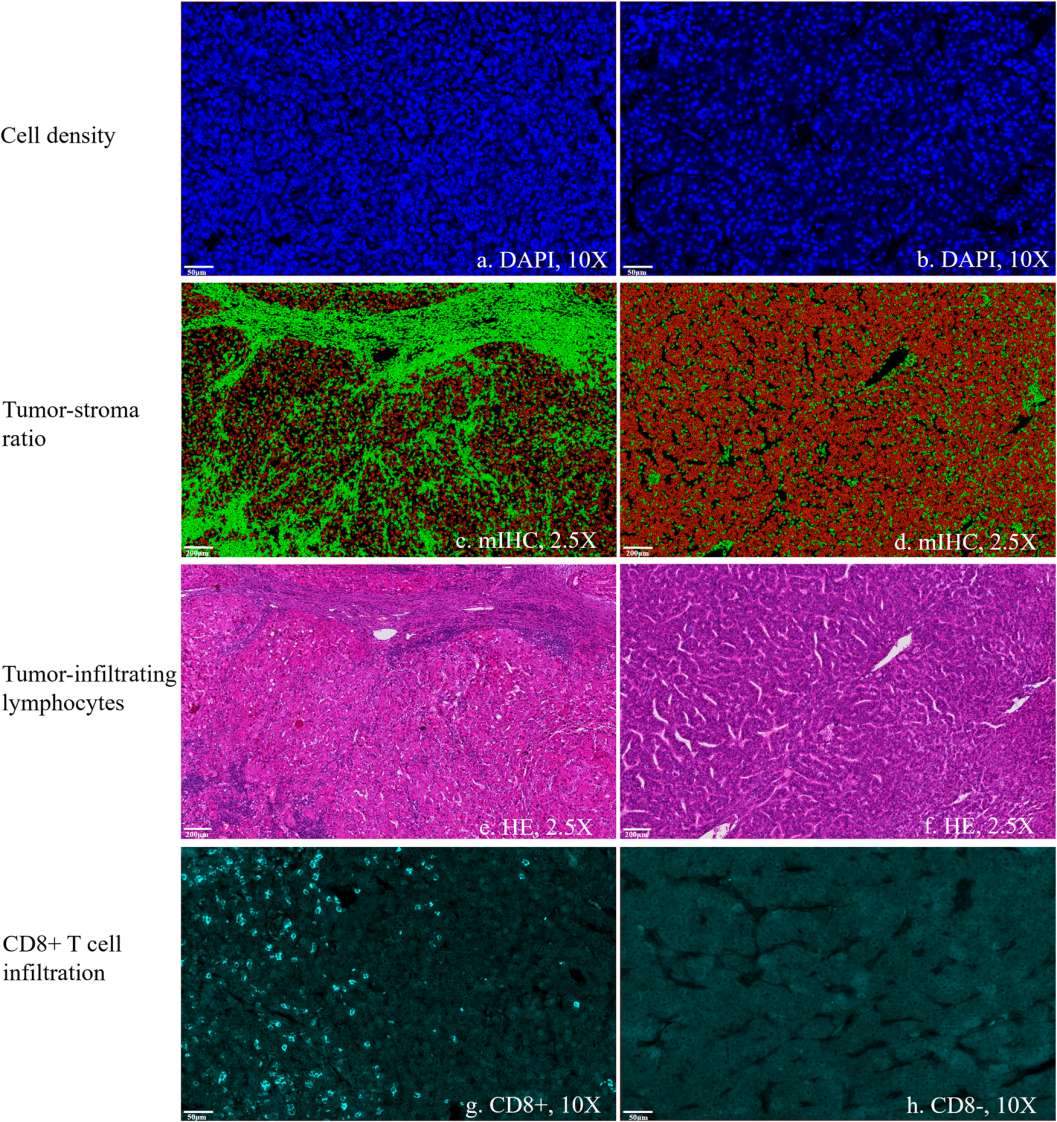
Cases of hepatocellular carcinoma (HCC) with different histopathological features. **a**, **b** HCC with high cell density (**a**) and low cell density (**b**) as assessed by the DAPI channel of mIHC. Blue dots are stained nuclei. **c**, **d** HCC with low tumor-stroma ratio (**c**) and high tumor-stroma ratio (**d**) according to cell classification on mIHC. Red represents tumor cells and green represents stroma cells. **e**, **f** Lymphocytes-rich HCC (**e**) and non-lymphocytes-rich HCC (**f**) as assessed by HE slides. **g**, **h** HCC with (**g**) and without (**h**) intra-tumoral CD8 + T cell infiltration

Inter-reader agreement was good regarding qualitative histopathological evaluation. The Kappa was 0.71 (0.49, 0.88) for the identification of LR-HCC and 0.88 (0.70, 0.99) for the identification of positive intra-tumoral CD8 + T cell infiltration. Figure 4 demonstrates the expression of each biomarker and their combined expression on the mIHC stain, along with the results of cell classification. Figure 5 presents examples of HCC cases with different histopathological features.

There were significant correlations between cell density and TSR (rho = 0.33, *p* = 0.005) and between TSR and LR-HCC (rho = −0.53, *p* < 0.001). No significant correlation was found between other histopathological features (Table 3).

**Table 3:** Associations between different tumor histopathological features of hepatocellular carcinoma

Histopathological features	Tumor-stroma ratio	Lymphocytes-rich HCC	CD8 + T cell infiltration
Cell density	**0.33 (0.005)**	−0.16 (0.17)	−0.11 (0.35)
Tumor-stroma ratio		**−0.53 (< 0.001)**	−0.17 (0.16)
Lymphocytes-rich HCC			0.11 (0.34)

Data in parenthesis are *p* values, and significant correlations are given in boldface

### Association between ADC value and histopathological features

At univariable analysis, cell density was the only histopathological feature associated with tumor ADC (rho = −0.39, *p* = 0.001). No significant correlation between tumor ADC and TSR (rho = −0.13, *p* = 0.29), LR-HCC (rho = 0.11, *p* = 0.37), intra-tumoral CD8 + T cell infiltration (rho = 0.04, *p* = 0.71), peri-tumor inflammation (rho = 0.12, *p* = 0.31), and peri-tumor fibrosis (rho = 0.05, *p* = 0.68) was found. After adjustment for cell density in the multivariable linear regression, the relationship between TSR and tumor ADC was still non-significant (Estimate = 0.001, *p* = 0.93, Table 4).

**Table 4:** Results of multivariable linear regression with MRI measurements as the dependent variable

Covariates	Estimate	Likelihood Chi-square	*p* value^#^
Factors associated with tumor ADC			
Cell density	−0.03	0.11	0.006
Tumor-stroma ratio	0.001	< 0.001	0.934
Factors associated with tumor stiffness			
Cell density	−0.03	0.004	0.607
Tumor-stroma ratio	−0.18	0.07	0.033
Tumor size	0.12	0.05	0.084
Factors associated with peri-tumor stiffness			
Intra-tumoral CD8 + T cell infiltration	0.63	0.08	0.021
Fibrosis stage	0.43	0.21	< 0.001
Age	0.04	0.28	< 0.001

*ADC* apparent diffusion coefficient

^#^ *p* value is for the test of the estimate

The peri-tumor ADC showed a weak negative correlation with the grade of peri-tumor inflammation (rho = −0.25, *p* = 0.04). However, it did not show a significant correlation with the stage of peri-tumor fibrosis (rho = −0.10, *p* = 0.42) or with any of the intra-tumoral histopathological features investigated, including cell density (rho = −0.04, *p* = 0.76), TSR (rho = 0.03, *p* = 0.84), LR-HCC (rho = 0.05, *p* = 0.70), and CD8 + T cell infiltration (rho = 0.04, *p* = 0.76).

### Association between stiffness and histopathological features

At univariable analysis, TSR (rho = −0.33, *p* = 0.005), LR-HCC (rho = 0.28, *p* = 0.02), and tumor size (rho = 0.40, *p* = 0.001) were significant factors associated with tumor stiffness. The tumor stiffness in LR-HCC was significantly higher than that in non LR-HCC (5.40 ± 1.65 Kpa vs. 4.33 ± 1.40 Kpa, *p* = 0.02). Since tumor-infiltrating lymphocytes were also counted as stroma cells when calculating the TSR, an intrinsic collinearity was expected between TSR and LR-HCC (rho = −0.53, *p* < 0.001). Therefore, only TSR and tumor size were included in the multivariable linear regression, where TSR was a significant factor influencing tumor stiffness (Estimate = −0.18, *p* = 0.03, Table 4). After adjustment for TSR and tumor size in the multivariable linear regression, the relationship between cell density and tumor stiffness remained non-significant (Estimate = −0.03, *p* = 0.61).

**Fig. 6:**
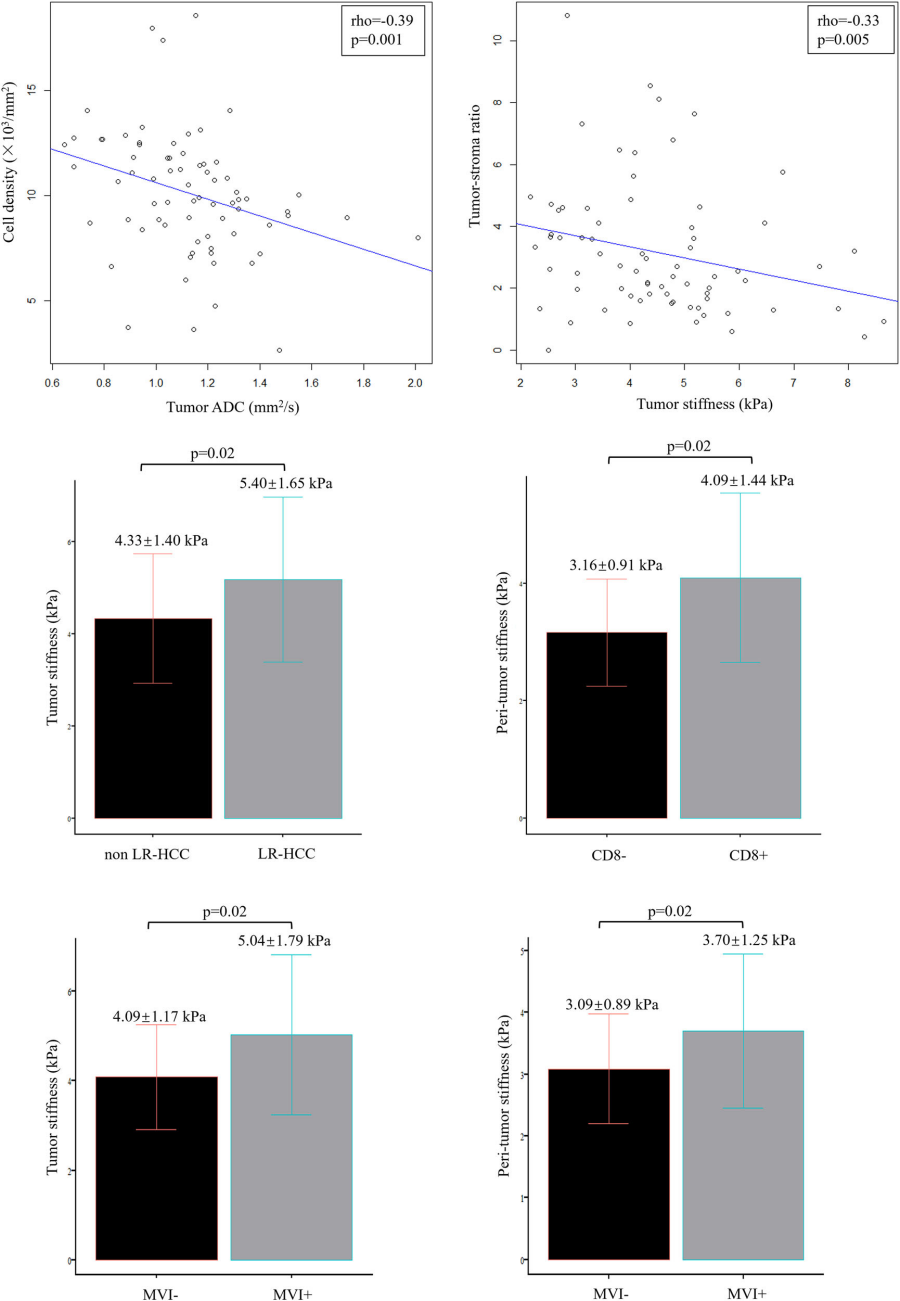
The associations between imaging biomarkers and histopathological features of hepatocellular carcinoma

Intra-tumoral CD8 + T cell infiltration (rho = 0.27, *p* = 0.02), fibrosis stage (rho = 0.39, *p* = 0.001), and age (rho = 0.41, *p* < 0.001) were significant variables associated with peri-tumor stiffness at univariable analysis. After adjustment for fibrosis stage (Estimate = 0.43, *p* < 0.001) and age (Estimate = 0.04, *p* < 0.001) in the multivariable linear regression, intra-tumoral CD8 + T cell infiltration remained a significant factor associated with peri-tumor stiffness (Estimate = 0.63, *p* = 0.02). The peri-tumor stiffness in HCC with CD8 + T cell infiltration was significantly higher than that in HCC without CD8 + T cell infiltration (4.09 ± 1.44 Kpa vs. 3.16 ± 0.91 Kpa, *p* = 0.02). The associations between MRI measurements and histopathological features are summarized in Table 4 and Fig. 6.

### Comparison of imaging and histopathological biomarkers between HCC with and without MVI

Tumor stiffness and peri-tumor stiffness were significantly higher in HCC with MVI. The mean tumor stiffness was 5.04 ± 1.79 kPa in HCC with MVI, and 4.09 ± 1.17 kPa in HCC without MVI. The mean peri-tumor stiffness was 3.70 ± 1.25 kPa in HCC with MVI, and 3.09 ± 0.89 in HCC without MVI. No significant difference was found in ADC measurements and histopathological biomarkers between groups. Table 5 summarizes the comparison of explored imaging and histopathological biomarkers between HCC with and without MVI.

**Table 5:** Comparison of imaging and histopathological biomarkers between hepatocellular carcinoma with and without microvascular invasion

	Without MVI (*N* = 41)	With MVI (*N* = 30)	*p*
Tumor ADC (mm^2^/s)	1.15 ± 0.22	1.09 ± 0.27	0.30
Tumor stiffness (kPa)	4.09 ± 1.17	5.04 ± 1.79	0.02
Peri-tumor ADC (mm^2^/s)	1.38 ± 0.28	1.29 ± 0.24	0.16
Peri-tumor stiffness (kPa)	3.09 ± 0.89	3.70 ± 1.25	0.02
Cell density (×10^3^/mm^2^)	10.38 ± 3.22	9.68 ± 2.52	0.33
Tumor-stroma ratio	2.94 ± 1.94	3.64 ± 2.99	0.23

*ADC* apparent diffusion coefficient, *MVI* microvascular invasion

## Discussion

This study investigated associations between ADC, stiffness, and several histopathological features of HCC. Results showed that ADC and stiffness were robust imaging biomarkers with excellent inter-reader agreement and were associated with different histopathologies of HCC. This analysis could provide further insights into the histopathological underpinnings of HCC and the role of MRI-derived imaging biomarkers in capturing these features.

The negative correlation between ADC and cell density in this study is consistent with prior conclusions [6, 21, 22], while the correlation between tumor stiffness and cell density was weak and non-significant. One potential explanation for the observed differences could be the higher spatial resolution of DWI compared to MRE imaging. Consequently, DWI may provide more detailed and accurate information regarding cellularity than MRE despite variations in physiological mechanisms. Previous studies have shown that both tumor ADC and stiffness can document the effects of cytoreductive treatment. For example, according to Kostek et al, advanced HCC patients with a favorable response to sorafenib had a significant increase in ADC value at the first radiological evaluation [23]. Qayyum et al found that changes in HCC stiffness at 6 weeks of immunotherapy correlated significantly with overall survival and time to disease progression [15]. Those findings suggested that changes in tumor ADC and stiffness might also serve as imaging biomarkers of treatment responders. Accordingly, for patients without these changes or with the opposite changes in corresponding imaging biomarkers, the treatment approach may need to be adjusted. Together with prior studies, the results of this study indicate that although tumor ADC and stiffness can both reflect tumor microstructure, ADC might be a better image biomarker for cellularity.

Tumor stroma is an important component of the tumor microenvironment, which provides favorable conditions for tumor growth and invasion [24, 25]. Recent studies have highlighted the role of stromal cells in modulating tumor immune status and its response to immunotherapy [25]. So far, only one study investigated the association between ADC and TSR as well as intra-tumoral lymphocytes in 53 biopsied HCCs [6]. Theoretically, increased stroma cells are associated with increased extracellular matrix deposition, which eventually leads to increased diffusion restriction (i.e., decreased ADC) [26]. Inconsistent with the weak correlation between ADC and TSR by prior study, our study found no significant correlation between tumor ADC and TSR. We speculate that this might be affected by the calculation of TSR on the cellular level, the lack of precise image-pathology match, and the use of different b values when calculating the ADC value (1000 s/mm^2^ in our study versus 600 s/mm^2^ in prior study). Nevertheless, tumor ADC continues to show a lack of association with intra-tumoral lymphocyte infiltration. On the other hand, it is well-known that the ADC value decreases with the increase of liver fibrosis stage [27]. However, the peri-tumor ADC did not correlate significantly with peri-tumor fibrosis in this study. This can be explained by the fact that most patients already had advanced fibrosis or cirrhosis at diagnosis. Under this situation, the peri-tumor ADC was more affected by other pathological conditions such as the grade of inflammation in this study.

To the best of our knowledge, this is the first study regarding associations between tumor stiffness and TSR in HCC. Gordic et al previously reported positive correlations between tumor stiffness and enhancement ratios [28], which might be partly explained by its correlation with TSR. Stroma-rich HCCs tend to have high microvascular density at pathology. The association between tumor stiffness and LR-HCC might also be explained by its association with TSR, given that tumor-infiltrating lymphocytes mainly present in tumor stroma. This result echoes with previous report about the strong positive correlation between HCC stiffness and intra-tumoral T lymphocytes in nine HCC biopsies [15]. However, there was only a weak and non-significant association between tumor stiffness and intra-tumoral CD8 + T cell infiltration, probably due to limited sample size. Interestingly, peri-tumor stiffness also showed a weak, yet significant, correlation with the intra-tumoral CD8 + T cell infiltration even after adjustment with other factors, indicating that the microenvironment remodeling of peri-tumor parenchyma is also involved in modulating the intra-tumoral immune status. This study suggested that tissue stiffness might be a promising image biomarker of intra-tumoral immune status of HCC. With meticulous evaluation in ongoing clinical trials, MRE might offer insights into identifying patients who could benefit from immunotherapy, potentially enabling the avoidance of unnecessary immunotherapy in non-responders.

Our study also showed that tumor ADC and stiffness correlated positively with peri-tumor ADC and stiffness, respectively. This observation supported the current conclusion in basic research regarding the interaction between tumor and peri-tumor microenvironments in the progression and treatment of HCC [29, 30]. According to Chen et al, both tumor stiffness and its stiffness difference between tumor and non-tumoral liver were significantly associated with tumor response to transarterial chemoembolization treatment [14]. Notably, no significant correlation was found between tumor ADC and tumor stiffness, suggesting that tumor ADC and stiffness were independent MRI biomarkers.

Our study does possess certain limitations. First, despite its prospective design, there was an absence of precise matching between the sites of histopathology and imaging analysis, which may have potentially weakened the association. second, the sample size of our study was limited, particularly in relation to the number of HCC cases with positive intra-tumoral CD8 + T cell infiltration. Third, due to limited sample size and follow-up time, we could not further validate the prognostic value of those imaging and histopathological biomarkers. Further studies are needed to verify the prognostic value of those biomarkers, especially the role of imaging biomarkers in patient stratification and monitoring treatment response.

In conclusion, ADC and stiffness are independent and robust image biomarkers that are associated with different microenvironment features of HCC. Tumor ADC surpasses tumor stiffness as a biomarker of cellularity. Tumor stiffness is associated with tumor-stroma ratio and peri-tumor stiffness might be an imaging biomarker of intra-tumoral immune microenvironment.

## Supplementary information

Electronic Supplementary Material

## Abbreviations

ADC: Apparent diffusion coefficient
BCLC stage: The Barcelona Clinic Liver Cancer stage
DWI: Diffusion-weighted imaging
HCC: Hepatocellular carcinoma
HE: Hematoxylin and eosin staining
ICC: Intraclass correlation coefficient
LR-HCC: Lymphocytes-rich hepatocellular carcinoma
mIHC: Multiplex immunohistochemical staining
MRE: Magnetic resonance elastography
MRI: Magnetic resonance imaging
MVI: Microvascular invasion
