# Supplementary material for: Apparent diffusion coefficient and tissue stiffness are associated with different tumor microenvironment features of hepatocellular carcinoma
Source: Eur Radiol. 2024 May 20;34(11):6980–91. doi: 10.1007/s00330-024-10743-2 (PMC11519246; doi:10.1007/s00330-024-10743-2)
Supplement: Supplementary file 1 — Electronic Supplementary Material [file 330_2024_10743_MOESM1_ESM.pdf]

Apparent diffusion coefficient and tissue stiffness are associated with different tumor microenvironment features of hepatocellular carcinoma

ELECTRONIC SUPPLEMENTARY MATERIAL

Supplementary methods for histopathological analysis

For mIHC staining, a panel of antibodies was utilized, including CD86 (Cell Signaling Technology, 76755SF), CD163 (Abcam, ab182422), CD8 (Abcam, ab237710), and α-smooth muscle actin (α-SMA) (Abcam, ab7817).

Cell classifications were conducted as follows: The pathologist (WZ, 10 years' experience in liver pathology) first reviewed the 5-channal mIHC slide and a free hands annotation was defined to include all available tumor area for quantitative analysis, excluding areas with poor quality (et cetera, any folded, blurred, missing or imperfect morphology). The watershed cell detection method was then applied to detect and segment all cells based on the nucleus stain by 4',6-diamidino-2-phenylindole (DAPI). After cell detection, the intensity features on all channels, cell shape features, and smoothed features at 50um radius were measured for all detected cells. By using the HE slides as reference, the same pathologist manually selected representative cells on the 5-channel mIHC slide to classify tumor cells (red) and stromal cells (green, including CD86+ or CD163+ macrophages, fibroblasts with or without α-SMA expression, and tumor-infiltrating lymphocytes), with the remaining cells set to "ignore". Based on these representative cells and calculated features, we used the built-in neural network classifier with 8 hidden layers (maximum iteration: 1000) for training to obtain cell classification. This pathologist supervised classifier was trained in multiple rounds of

optimization to produce the most satisfactory cell classification. The trained cell classifier was then applied to all whole slide image with tumor annotation via a built-in script in QuPath, and the annotation area (mm^2) and the number of each cell type were recorded.

Supplementary Table 1. The intraclass correlation coefficient of MRI measurements in hepatocellular carcinomas with and without necrosis, respectively.

	Tumor ADC	Tumor stiffness	Peri-tumor ADC	Peri-tumor stiffness
Without necrosis (n=50)	0.77 (0.66, 0.85)	0.99 (0.98, 0.99)	0.76 (0.68, 0.94)	0.93 (0.91, 0.96)
With necrosis (n=22)	0.86 (0.69, 0.89)	0.82 (0.58, 0.93)	0.74 (0.49, 0.95)	0.96 (0.90, 0.97)

Data in parenthesis are 95% confidence intervals of the intraclass correlation coefficient. ADC, apparent diffusion coefficient.

Supplementary Table 2. Comparison of MRI measurements between hepatocellular carcinomas with and without necrosis.

	Tumor ADC (sec/mm^2)	Tumor stiffness (kPa)	Peri-tumor ADC (sec/mm^2)	Peri-tumor stiffness (kPa)
Without necrosis (n=50)	1.12 ± 0.25	4.35 ± 1.51	1.34 ± 0.26	3.34 ± 1.18
With necrosis (n=22)	1.14 ± 0.22	4.94 ± 1.41	1.36 ± 0.29	3.34 ± 0.87
p value	0.780	0.131	0.739	0.999

Data are median value and data in parenthesis are interquartile ranges. ADC, apparent diffusion coefficient.

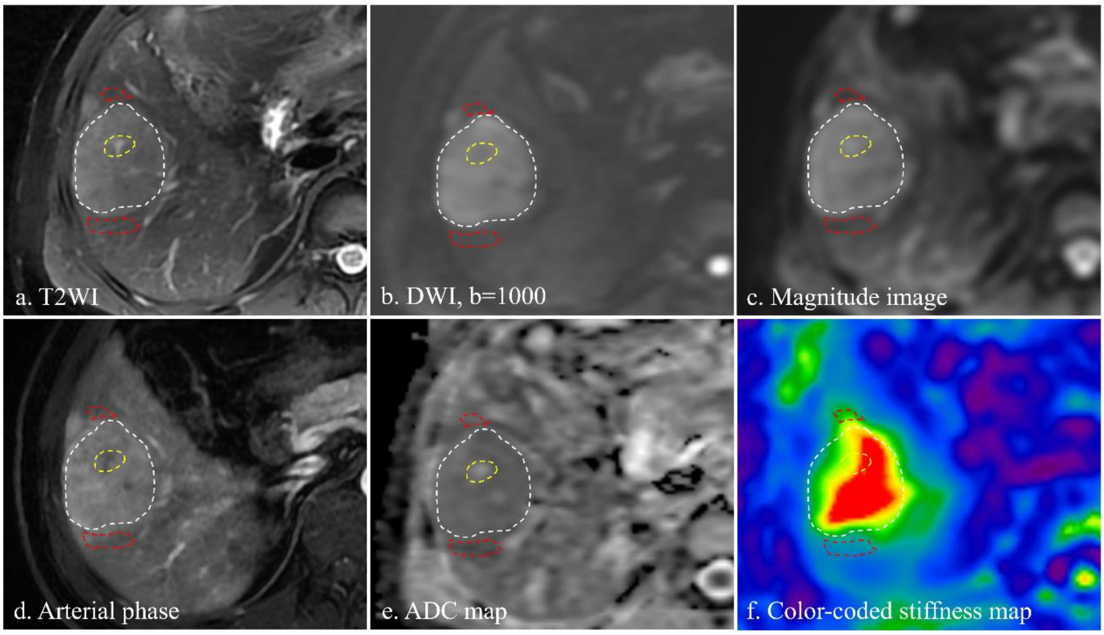

Supplementary Figure 1. The measurement of apparent diffusion coefficient (ADC) and stiffness in the tumor (white) and peri-tumor area (red), intratumoral necrosis was excluded from analysis (yellow). a. T2 weighted image; b. diffusion-weighted image; c. the T2-weighted magnitude image of magnetic resonance elastography; d. late arterial phase of dynamic contrast enhanced imaging; e. the ADC map; f. the color-coded stiffness map.

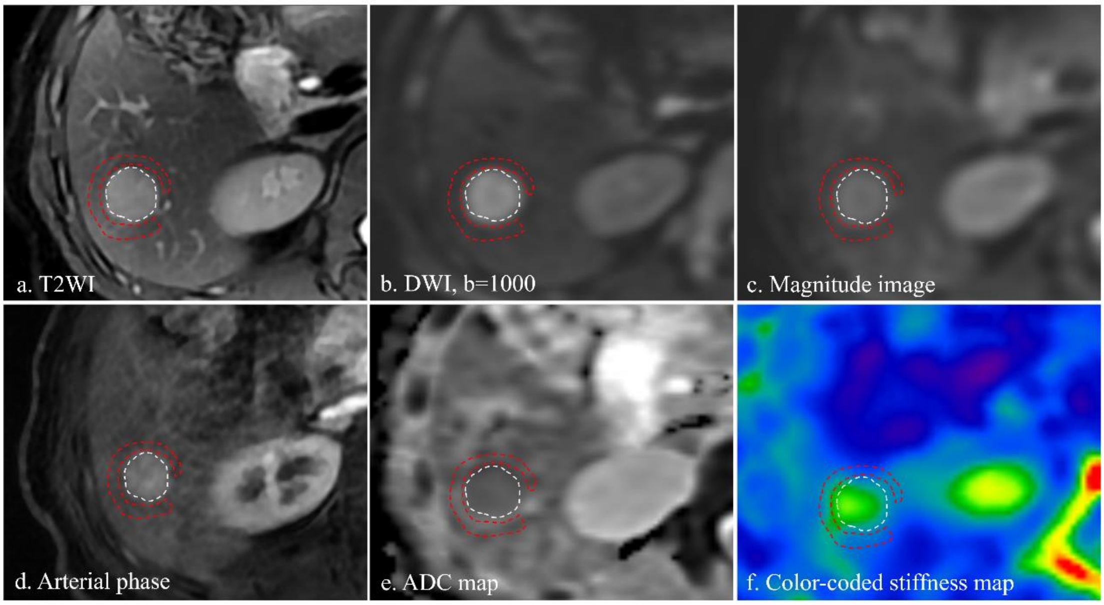

Supplementary Figure 2. The measurement of apparent diffusion coefficient (ADC) and stiffness in the tumor (white) and peri-tumor area (red). a. T2 weighted image; b. diffusion-weighted image; c. the T2-weighted magnitude image of magnetic resonance elastography; d. late arterial phase of dynamic contrast enhanced imaging; e. the ADC map; f. the color-coded stiffness map.

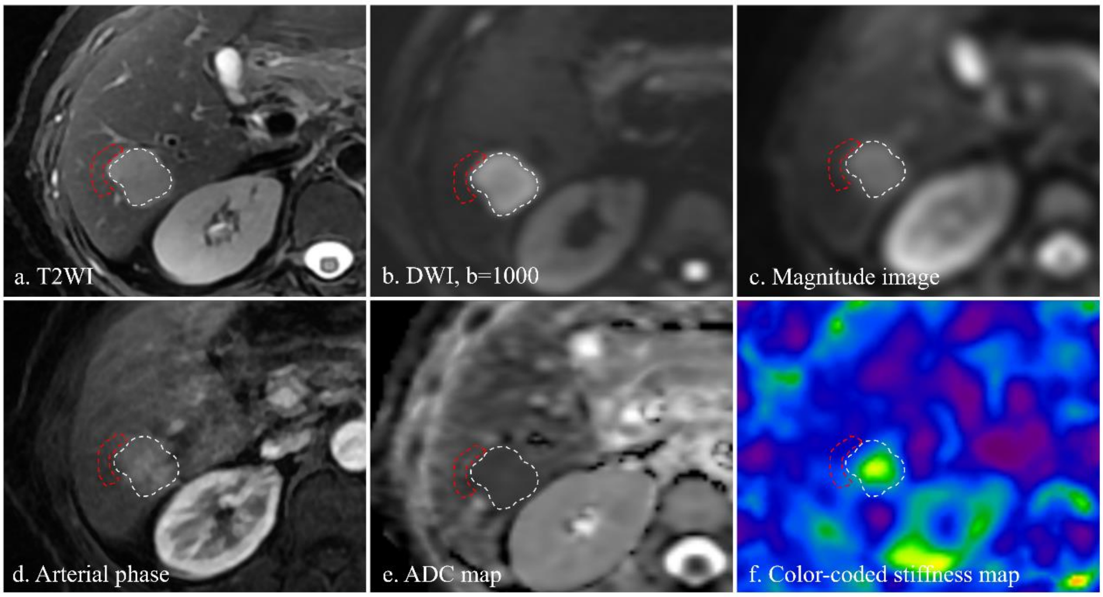

Supplementary Figure 3. The measurement of apparent diffusion coefficient (ADC) and stiffness in the tumor (white) and peri-tumor area (red). a. T2 weighted image; b. diffusion-weighted image; c. the T2-weighted magnitude image of magnetic resonance elastography; d. late arterial phase of dynamic contrast enhanced imaging; e. the ADC map; f. the color-coded stiffness map.
